# The Challenge of Integrating eHealth Into Health Care: Systematic Literature Review of the Donabedian Model of Structure, Process, and Outcome

**DOI:** 10.2196/27180

**Published:** 2021-05-10

**Authors:** Rosian Tossaint-Schoenmakers, Anke Versluis, Niels Chavannes, Esther Talboom-Kamp, Marise Kasteleyn

**Affiliations:** 1 Saltro Diagnostic Centre Utrecht Netherlands; 2 National eHealth Living Lab Leiden University Medical Centre Leiden Netherlands; 3 Public Health and Primary Care Department Leiden University Medical Centre Leiden Netherlands; 4 Unilabs Group Geneva Switzerland

**Keywords:** eHealth, digital health, blended care, quality, integration, health care organization, structure, process, outcome

## Abstract

**Background:**

Health care organizations are increasingly working with eHealth. However, the integration of eHealth into regular health care is challenging. It requires organizations to change the way they work and their structure and care processes to be adapted to ensure that eHealth supports the attainment of the desired outcomes.

**Objective:**

The aims of this study are to investigate whether there are identifiable indicators in the structure, process, and outcome categories that are related to the successful integration of eHealth in regular health care, as well as to investigate which indicators of structure and process are related to outcome indicators.

**Methods:**

A systematic literature review was conducted using the Donabedian Structure-Process-Outcome (SPO) framework to identify indicators that are related to the integration of eHealth into health care organizations. Data extraction sheets were designed to provide an overview of the study characteristics, eHealth characteristics, and indicators. The extracted indicators were organized into themes and subthemes of the structure, process, and outcome categories.

**Results:**

Eleven studies were included, covering a variety of study designs, diseases, and eHealth tools. All studies identified structure, process, and outcome indicators that were potentially related to the integration of eHealth. The number of indicators found in the structure, process, and outcome categories was 175, 84, and 88, respectively. The themes with the most-noted indicators and their mutual interaction were inner setting (51 indicators, 16 interactions), care receiver (40 indicators, 11 interactions), and technology (38 indicators, 12 interactions)—all within the structure category; health care actions (38 indicators, 15 interactions) within the process category; and efficiency (30 indicators, 15 interactions) within the outcome category. In-depth examination identified four most-reported indicators, namely “deployment of human resources” (n=11), in the inner setting theme within the structure category; “ease of use” (n=16) and “technical issue” (n=10), both in the technology theme within the structure category; and “health logistics” (n=26), in the efficiency theme within the outcome category.

**Conclusions:**

Three principles are important for the successful integration of eHealth into health care. First, the role of the care receiver needs to be incorporated into the organizational structure and daily care process. Second, the technology must be well attuned to the organizational structure and daily care process. Third, the deployment of human resources to the daily care processes needs to be aligned with the desired end results. Not adhering to these points could negatively affect the organization, daily process, or the end results.

## Introduction

Health care is changing, whereby patient empowerment, democratization of the internet, and an increasing burden on health care professionals play influential roles [1-3]. In line with these trends, innovations such as eHealth are required to maintain high quality of care [4-6]. eHealth includes a wide range of web-based interventions, for example e-consults, telemonitoring, and web-based viewing of medical records [1,7,8]. However, eHealth is more than a technology; it is another way of working and thinking and requires a change in attitude, which goes beyond the boundaries of a local health care organization [9,10].

The most comprehensive definition of eHealth with reference to the organizational context is that provided by Eysenbach [11]:

e-health is an emerging field in the intersection of medical informatics, public health and business, referring to health services and information delivered or enhanced through the Internet and related technologies. In a broader sense, the term characterizes not only a technical development, but also a state-of-mind, a way of thinking, an attitude, and a commitment for networked, global thinking, to improve health care locally, regionally, and worldwide by using information and communication technology.

In other words, the integration of eHealth into traditional health care requires organizational and behavioral changes for both health care professionals and patients [9,10].

Organizations are increasingly working with eHealth; however, implementing eHealth into the regular health care system requires organizations to change the way they work [9-11]. eHealth enables patients to have a more active role in managing their health [7,12,13], which affects interactions between the patient and health care professional [14-17]. Furthermore, working with eHealth technology requires workflow adjustments for health care professionals [18,19]. The organization’s structure and care processes need to be adapted to ensure that eHealth supports the attainment of desired outcomes [20,21].

The challenge of optimally integrating eHealth into health care is thus a complex organizational issue. Several studies have identified elements to promote eHealth adoption, such as the degree of complexity, adaptability of the technology, costs, and stakeholder value [20,22], but uncertainty remains on how digital and traditional health care can blend successfully in the long term. With different definitions of eHealth available in the literature [10,11,23], and unclear barriers for facilitators in the application of eHealth [19], there is a need for further research on how eHealth can successfully be integrated into health care.

The aim of this study is to analyze how the integration of eHealth can be organized optimally by reviewing studies evaluating real-world eHealth interventions. The Donabedian framework of Structure-Process-Outcome (SPO) [24] was used, allowing the identification of relevant indicators that demonstrate how effective the integration of eHealth is in the organization.

**Figure 1:**
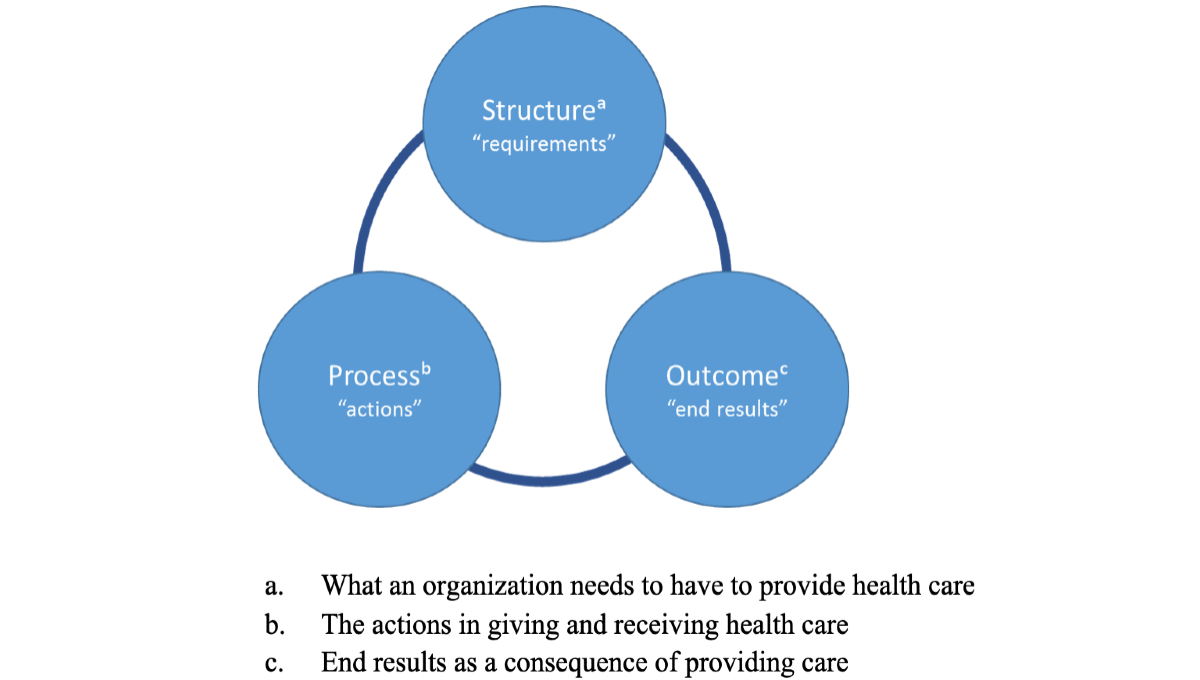
Donabedian Structure-Process-Outcome framework.

According to the Donabedian model, the quality of health care can be assessed by three components that are relevant for organizations: structure (ie, requirements of the organization), process (ie, actions to be taken), and outcome (ie, end results), as shown in Figure 1 [24,25]. *Structure* is defined as the setting in which health care is provided (eg, facilities, equipment, numbers, and qualification of personnel); *process*, as what is actually done in giving and receiving care (eg, patient and doctor activities, doctor-patient communication and information); and *outcome,* as the consequence of the provided health care (eg, health status, satisfaction, and costs) [24-26]. Quality of health care is based on different aspects of these three categories and their relationships. As Donabedian eloquently puts it: “A good structure increases the likelihood of good process, and good process increases the likelihood of good outcomes” [24]. The interaction between the categories can be bidirectional, and it is not a simple separation between cause and effect [25]. The movement is an “unbroken chain of antecedents, followed by intermediate ends, which are themselves the means to still further ends” [25].

The aim of this systematic review is twofold: (1) to investigate whether there are identifiable indicators in the structure, process, and outcome categories related to the successful integration of eHealth in regular health care and (2) to investigate which indicators of structure and process are related to outcome indicators.

## Methods

### Theoretical Framework

**Figure 2:**
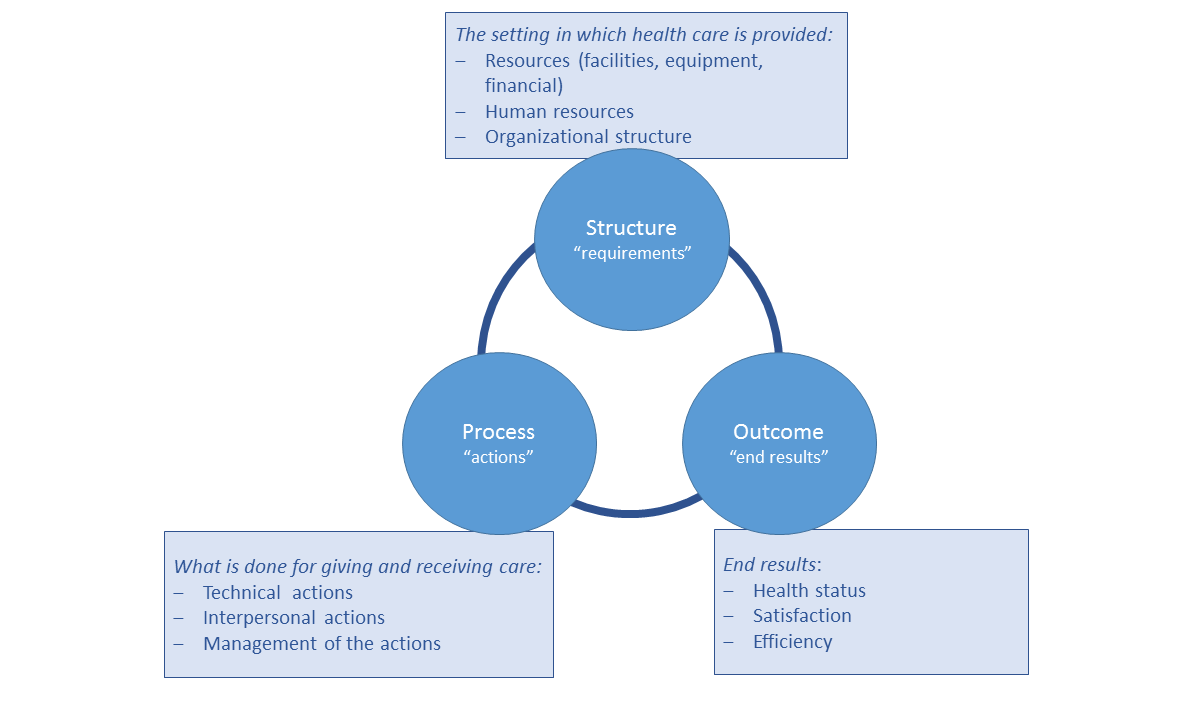
Explanation of the Structure-Process-Outcome categories of the Donabedian model.

The Donabedian SPO framework was used to identify the indicators of structure, process, and outcome that potentially affect the integration of eHealth into health care organizations. The Donabedian framework covers all relevant aspects of an organization’s structure, process, and outcome and their interrelations, and it combines these aspects with health and social factors. Therefore, it is a suitable model to evaluate the organization of eHealth within health care organizations. The SPO categories are thematically explained in Figure 2 [24,25].

The Donabedian SPO framework was designed in the 20th century before the introduction of eHealth. For this review, the SPO framework was adjusted to be compatible with the current time and incorporated the application of eHealth. The adjustments are described in the themes presented in Textbox 1.

**Figure 3:**
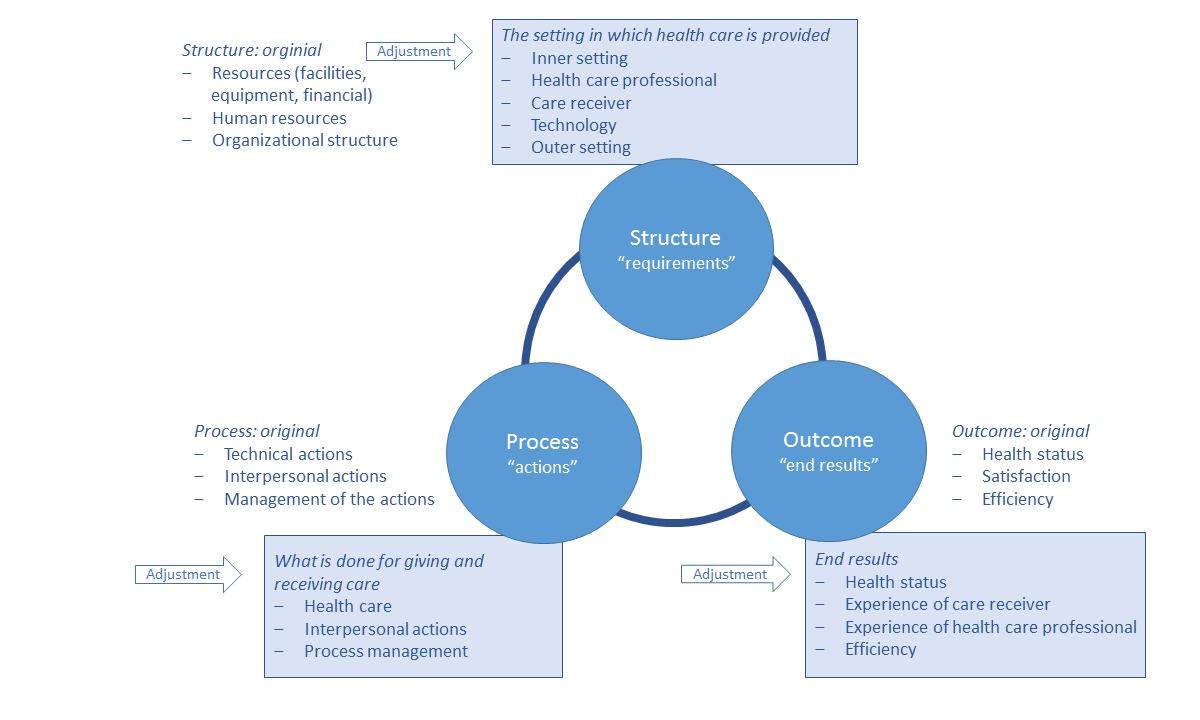
Adjustment to the themes of the Structure-Process-Outcome framework, considering eHealth integration.

The adjustments to the SPO framework are shown in Figure 3.

**Adjustment of the Structure-Process-Outcome framework into themes, to integrate the application of eHealth into the health care system.**

- **Structure**: The *setting* of provided care can be internal and/or external. Therefore, a distinction was made between *inner* and *outer settings*. With regard to *resources*, *technology* was added as a separate theme to cover eHealth. This was done because the focus of this research was eHealth. The remaining parts of the *resources* are covered under inner setting. *Human resources*, besides *health care professionals*, included *care receivers.* Their mutual involvement is required and is therefore also considered a conditional human resource [1]. *Organizational structure* was split into *inner setting* and *outer setting*, in line with the reasons given above, and to take the external stakeholders into account [27].
- **Process**: Instead of *technical actions*, the term *health care actions* was used, to avoid confusion with the term *technology* in the structure. *Interpersonal actions* remained unchanged. *Management of the actions* was shortened to *process management*.
- **Outcome**: *Health status* was retained as *health status*. *Satisfaction* was broadened to include *experience of the health care receiver* and *experience of the health care provider*, as both are pivotal outcome parameters in the *health care* process [28,29]. *Efficiency* remained unchanged.

### Search Strategy

This systematic review followed the PRISMA (Preferred Reporting Items for Systematic Reviews and Meta-analyses) guidelines. The research question was as follows: “How are structure indicators and/or process indicators related to eHealth or blended health care outcome indicators?”

Two authors (RT and MK) searched PubMed, EMBASE, Web of Science, Cochrane, and Emcare databases for relevant studies published up to December 12, 2019. They searched for the following terms in the titles, abstracts, and keywords of the published papers: structure* indicators* or process* indicators* or outcomes* indicators* and [blended care or eHealth* or telehealth*]. Multimedia Appendix 1 contains the full search details. After the search, two authors (RT and AV) screened the titles and abstracts of the relevant articles. Studies were included if they mentioned (1) the use of eHealth or blended care for diagnostics or treatment and (2) structure, process, or outcome indicators. Quantitative, qualitative, and mixed method study designs were included. A study was excluded if (1) it was a protocol, review, meta-analysis, grey literature, book chapter, oral presentation, or poster presentation; (2) it was published in a language other than English or Dutch; (3) full-text of the article was not available; (4) the intervention was not implemented (eg, conducted research regarding the users’ expectations towards a prototype); or (5) the intervention used an analog application via plain-old-telephone lines. Of the remaining articles, RT reviewed the full texts. To ensure reliability, AV randomly selected about 10% of the fully reviewed articles for a blind review. Discrepancies were resolved by discussion. In case of uncertainty, a third author (MK) was consulted.

### Data Extraction

Data extraction sheets were designed to provide an overview of the (1) study characteristics (eg, title, author, study aim, setting, disease, and quality appraisal); (2) characteristics of the eHealth intervention (eg, technology and function) and description of the intervention; (3) distribution of indicators into themes and categories related to the integration of eHealth into health care; and (4) interaction among the indicators, presented as themes.

RT designed the first concepts of the data extraction sheets. Authors RT, MK, NC, and ET discussed the design of the data extraction sheets to ensure their usability. Improved sheets were developed accordingly. The blind reviewer (AV) did not discuss the data extraction sheets. The included articles were reread by RT to check whether data clustering was complete and logical and for purposes of data pooling itself. AV selected a sample of 10% of the included articles for data extraction. Discrepancies were resolved by discussion.

### Quality Appraisal

The Mixed Methods Appraisal Tool (MMAT) was used to appraise the quality of eligible studies in mixed methods systematic reviews—that is, reviews that included qualitative research, randomized controlled trials, nonrandomized studies, quantitative descriptive studies, and mixed methods studies [30]. The MMAT allows determination of the quality of different empirical study designs by using the same measure of five criteria in the chosen category. MMAT scores range on a scale of 1 to 5, with 1 indicating the lowest quality and 5 indicating the highest quality.

### Classification of eHealth Interventions

eHealth interventions were ordered by type of technology and functionality. For technology, the classification proposed by Nictiz was used, distinguishing websites, apps, video communication, sensors, and wearables, domotics, robotics, and big data (ie, artificial intelligence) [31]. This classification is based on international studies [10,32]. For the present study, eHealth only concerns digital interventions and not analog ones such as analog applications via plain-old-telephone lines; this is in line with the classification proposed by Nictiz. For labelling the functionality, the second and third tiers of the National Institute for Health and Care Excellence (NICE) [33] were used, because these functionalities measure patient outcomes (Tier one consists of system services with no measurable patient outcomes). The functions were classified as communication, self-management, clinical calculation, active monitoring, diagnosis, and treatment [33].

### Organization of Indicators and (Sub)themes of the SPO Framework

Indicators that had a potential impact on the integration of eHealth in health care were extracted and organized by the relevant theme according to the adjusted SPO framework (Textbox 1). In addition, the reported interactions between the indicators were extracted and organized by the relevant categories and themes. For a clear overview, the indicators within each theme were further divided into two subthemes by RT and ET (Table 1). The creation of subthemes was an iterative process. When reading the full texts, we found some definitions that sharpened some of the subthemes. The full definitions of the themes and subthemes are provided in Multimedia Appendix 2.

For each of the extracted indicators, the relevant impact on the integration of eHealth was noted. As there is no general standard for when eHealth is successful or effective [3,19], nor did the included studies specify such standards, these indicators were labeled as *advantage*, *disadvantage*, or *neutral*. An advantage in the structure and process categories indicates a positive effect on the integration and/or a positive effect on the outcome. A disadvantage in the structure and process categories indicates a negative effect on the integration and/or a negative effect on the outcome. An indicator that did not turn out to be an advantage nor a disadvantage was labeled neutral. The extracted indicators were noted as *advantage*, *disadvantage*, or *neutral*, in line with the evaluation performed in the corresponding study.

The following results are presented in this paper: (1) distribution of the indicators into (sub)themes and categories, and the impact on the integration of eHealth into health care; (2) most frequently reported indicators (ie, reported 10 times or more); and (3) interaction among indicators organized into themes and categories.

**Table 1:** Themes and subthemes in the structure, process, and outcome categories.

Category and theme			Subtheme
**Structure**			
	Inner setting	Support of primary process Culture and leadership	
	Health care professional	Skills Attitude	
	Care receiver	Daily life Baseline characteristics	
	Technology	Usability and functionality Interaction with electronic health record	
	Outer setting	Finance and legislation Involvement of stakeholders	
**Process**			
	Health care actions	Workflow Patient-centered	
	Interpersonal actions	Personal Shifting roles	
	Process management	Quality improvement Mistake-proofing	
**Outcome**			
	Health status	Clinical or functional Intrapersonal	
	Experience of care recipient	Satisfaction Convenience	
	Experience of health care professional	“What’s in it for me” “What’s in it for them”	
	Efficiency	Operations Revenues	

## Results

### Study Selection

**Figure 4:**
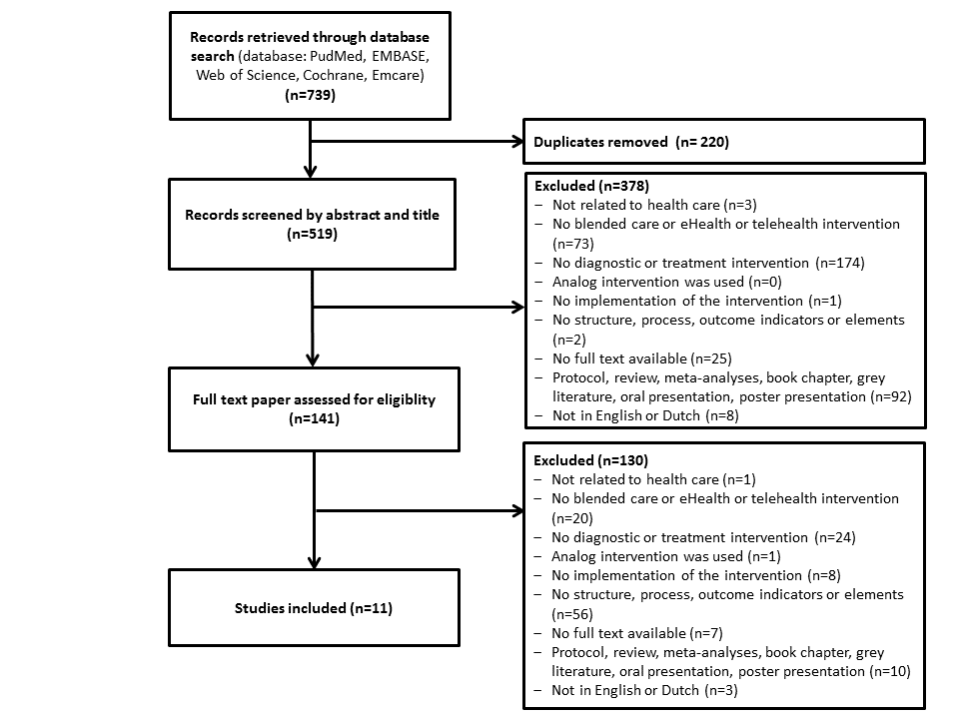
Flowchart of the systematic review.

The systematic search led to the identification of 11 eligible articles, selected from a total of 739 articles shortlisted initially (Figure 4).

### Data Results: Study and eHealth Characteristics

#### Study Characteristics

The included studies cover various study designs, diseases, and health care settings. Most studies were published after 2017 [27,34-41] and were of high quality [27,34,36,37,39,40,42]. Table 2 shows a detailed description of the characteristics of the included studies.

**Table 2:** Study characteristics.

Title, author (year)	Aim of the study	Study design	Setting and country	Disease	Sample size (N) and participant type	Quality of study^a^
Implementation of the blended care self-management program for caregivers of people with early-stage dementia, Boots et al (2017) [34]	To assess the internal and external validity of the trial and its implementation to inform effect analysis	Mixed methods, nested in an RCT^b^	Elderly care, home setting, the Netherlands	Early-stage dementia	N=62; Informal caregivers, psychologists, nurses	4.5
Lack of adoption of a mobile app to support patient self-management of diabetes and hypertension in a Federally Qualified Health Centre, Thies et al (2017) [35]	To understand why the trial was unsuccessful	Qualitative (interview) analysis	Primary care, USA	Uncontrolled diabetes and hypertension	N=13; Patients, primary care provider, nurses, research assistants	3.0
“Sounds a bit crazy, but it was almost more personal:” A qualitative study of patient and clinician experiences of physical therapist-prescribed exercise for knee osteoarthritis via skype, Hinman et al (2017) [36]	To explore the experience of therapists and patients using Skype for exercise management of knee osteoarthritis	Qualitative study, nested in an RCT	Rehabilitation at home, Australia	Knee osteoarthritis	N=20; Patients, physical therapists	5.0
The challenge of real-world implementation of web-based share care software, Lycett et al (2014) [43]	To highlight the challenges of implementing software and reporting on the extent to which the software met its implementations and user aims	Mixed methods study, nested within an RCT	Children’s Hospital, General Practices, Australia	Obesity	N=27; General Practitioners	2.5
Implementation of a multicomponent telemonitoring intervention to improve nutritional status of community-dwelling older adults, Van Doorn-van Atten et al (2018) [37]	To study how PhysioDom Home Dietary Intake Monitoring was delivered and received by participants and nurses, and to study the intervention’s mechanism of impact	Mixed methods study	Home care and/or lived in a service flat in sheltered accommodation, the Netherlands	At risk of undernutrition	N=105; Patients, nurses	4.5
Implementation of internet-delivered cognitive behaviour therapy within community mental health clinics, Hadjistavropoulos et al (2017) [27]	To understand facilitators and barriers impacting the uptake and implementation of internet cognitive behavior therapy	Mixed methods study	Community Mental Health Clinic, Canada	Depression and anxiety	N=33; Therapists, managers	4.5
Implementation and evaluation of the safety net specialty care program in the Denver Metropolitan Area, Fort et al (2017) [38]	To describe the program, identify aspects that work well, areas for improvement, and offer lessons learned	Mixed methods study	Safety-net: a non-profit integrated health care system, USA	Uninsured patients	N=43; Patients, primary care clinicians, specialists	3.5
Perceived improvement in integrated management of childhood illness implementation through use of mobile technology, Mitchell et al (2012) [42]	To examine health care provider and carer perceptions of electronic Integrated Management of Childhood Illness (eIMCI) in diagnosing and treating childhood illnesses	Qualitative study (semi-structured interviews)	Health centers, Tanzania	Childhood illness in children 5 years or younger	N=31; Carers, health care providers	4.5
High level of integration in integrated disease management leads to higher usage in the e-Vita Study, Talboom-Kamp et al (2017) [39]	To analyze the factors that successfully promote the use of a self-management platform for chronic obstructive pulmonary disease patients	Quantitative, nonrandomized, parallel cohort design	Primary care, the Netherlands	Chronic obstructive pulmonary disease	N=215; Patients	4.0
What drives adoption of a computerised, multifaceted quality improvement intervention for cardiovascular disease management in primary healthcare settings? Patel et al (2018) [40]	To identify and explain the underlying mechanisms by which the intervention did and did not have an impact	Mixed methods study	Primary care, Australia	Cardiovascular disease	N=19; Patients, general practitioners, nurses, aboriginal health workers	4.5
Exploring the challenges of implementing a web-based telemonitoring strategy for teenagers with inflammatory bowel disease, Dijkstra et al (2019) [41]	To evaluate whether the telemonitoring strategy could move from a demonstration project to one that is sustained within existing sites	Mixed methods study, nested within an RCT	Pediatric gastroenterology centers, the Netherlands	Inflammatory bowel disease	N=27; Researchers, clinicians, hospital decision makers, web designer	2.5

^a^Methodological quality of studies assessed with MMAT, ranging from 0 (lowest) to 5 (highest).

^b^RCT: randomized controlled trial.

#### eHealth Intervention Characteristics, Descriptions, and Results

The most frequently used digital technology was websites (n=7) [27,34,37-41], and the most frequently reported functions [33] of the technology were self-management (n=6) [34-37,39,41] and communication (n=6) [35,37,39-41,43]. Table 3 shows an overview of the eHealth intervention characteristics, descriptions, and the study results. A detailed description of indicators, sorted according to the structure, process, outcome categories and their respective (sub)themes, are highlighted in the next paragraph.

**Table 3:** eHealth intervention characteristics, descriptions of the intervention, and study results.

First author	Technology; intervention name	eHealth function	Intervention	Study results (findings)^a^
Boots [34]	Website; Partner in Balance	Self-management	Face-to-face coaching with tailored web-based modules.	The participation rate of eligible caregivers was 51.9% (80/154). Recruitment barriers included lack of computer and need for support. Young age and employment were considered recruitment facilitators. All coaches attended training and supervision in blended care self-management. Deviations from the structured protocol were reported on intervention time, structure, and feedback. Coaches described an intensified relationship with the caregiver post-intervention. Caregivers appreciated the tailored content and positive feedback. The blended structure increased their openness. Overall, personal goals were attained after the program (*t*>50). Implementation barriers included lack of financing, time, and deviating target population.
Thies [35]	App; Undisclosed	Self-management, communication	Platform for active collaboration between patients and their primary care teams.	There was a poor fit between the app, end-users, and recruitment and treatment approaches in the setting. Usability testing might have revealed this prior to launch, but this was not an option. There was not sufficient time during routine care for clinical staff to familiarize patients with the app or to check clinical data and messages, which are unreimbursed activities. Some patients did not use the app appropriately. The lack of integration with the electronic health record was cited as a problem for both patients and staff who also said the app was just one more thing to attend to.
Hinman [36]	Video communication; Telerehabilitation via Skype	Self-management, treatment	Individualized home-based training strengthening program via Skype delivered physiotherapy.	Six themes arose from both patients and therapists. The themes were Structure: technology (ease of use, variable quality, set-up assistance helpful) and patient convenience (time-efficient, flexible, increased access); Process: empowerment to self-manage (facilitated by home environment and therapists focusing on effective treatment) and positive therapeutic relationships (personal undivided attention from therapists, supportive friendly interactions); and Outcomes: satisfaction with care (satisfying, enjoyable, patients would recommend, therapists felt Skype more useful as adjunct to usual practice) and patient benefits (reduced pain, improved function, improved confidence and self-efficacy). A seventh theme arose from therapists regarding process: adjusting routine treatment (need to modify habits, discomfort without hands-on, supported by research environment).
Lycett [43]	Website, HIE^b^; Shared-Care Obesity Trial in Children (HOPSCOTCH)	Communicate	Children attended a tertiary appointment with a pediatrician and dietician specializing in childhood obesity, followed-up by general practitioner consultations over the following year, supported by shared-care web-based software.	Software implementation posed difficult and at times disabling technological barriers. The software’s speed and inability to seamlessly link with day-to-day software was a source of considerable frustration. Overall, general practitioners rated software usability as poor, although most (68%) felt that the structure and functionality of the software was useful.
Van Doorn-van Atten [37]	Website; PhysioDom HDIM^c^	Self-management, communication	Nutritional telemonitoring, education, a follow-up of telemonitoring measurement by a nurse.	About 80% of participants completed the intervention. Drop-outs were significantly older, had worse cognitive and physical functioning, and were more care-dependent. The intervention was largely implemented as intended and was received well by participants, but less well by nurses. Participants adhered better to weight telemonitoring than to telemonitoring by means of questionnaires, for which half the participants needed help. Intention to use the intervention was predicted by performance expectancy and social influence. No association was found between process indicators and intervention outcomes.
Hadjistavropoulos [27]	Website; ICBT^d^	Treatment	Web-based lessons that provide psychoeducation and instructions and therapist support via email or telephone.	ICBT implementation was perceived to be most prominently facilitated by intervention characteristics (namely, the relative advantages of ICBT compared to face-to-face therapy, the quality of the ICBT program that was delivered, and evidence supporting ICBT) and implementation processes (namely the use of an external facilitation unit that aided with engaging patients, therapists, and managers and ICBT implementation). The inner setting was identified as the most significant barrier to implementation as a result of limited resources for ICBT combined with greater priority given to face-to-face care.
Fort [38]	Website; Safety Net Specialty Care Program	Diagnosis, treatment	E-consults between primary care clinicians and specialist, face-to-face visits to the patients from a specialist, and continuing medical education for the primary care clinicians.	In the first 20 months of the program, safety-net clinicians at 23 clinics made 602 e-consults to specialists, and 81 patients received face-to-face specialist visits. Of 204 primary care clinicians, 103 made e-consults; 65 specialists participated in the program. Aspects facilitating program use were referral case managers' involvement and the use of clear, concise questions in e-consults. Key recommendations for process improvement were to promote an understanding of the different healthcare contexts, support provider-to-provider communication, facilitate hand-offs between settings, and clarify program scope.
Mitchell [42]	App; Electronic Integrated Management of Childhood Illness (eIMCI)	Diagnosis, treatment	An electronic handheld device or personal digital assistant, to guide the health care provider through IMCI protocols.	Providers expressed positive opinions on eIMCI, noting that the personal digital assistants were faster and easier to use than were the paper forms and encouraged adherence to IMCI procedures. Carers also held a positive view of eIMCI, noting improved service from providers, a more thorough examination of their child, and a perception that providers who used the personal digital assistants were more knowledgeable.
Talboom-Kamp [39]	Website; e-Vita COPD^e^	Self-management, communication	Insight into personal health data, self-monitoring of health values, education, and a coach for attaining personal goals.	Use of a self-management platform was higher when participants received adequate personal assistance about how to use the platform. Blended care, where digital health and usual care are integrated, will likely lead to increased use of the web-based program.
Patel [40]	HIE, website; HealthTracker	Communication, monitoring	Real-time decision support integrated with electronic medical records; CVD^f^ risk communication tool between provider and patient; clinical audit tool; web portal providing peer-ranked performance trends.	A complex interaction was found between implementation processes and several contextual factors affecting uptake of the intervention. There was no clear association between team climate, job satisfaction, and intervention outcomes. There were four spheres of influence that appeared to enhance or detract from normalization of the intervention: organizational mission and history, leadership, team environment, and technical integrity of the intervention.
Dijkstra [41]	Website, IBD-live	Monitoring, self-management, communication	Flarometer, platform for direct communication with the IBD^g^ team, module with study questionnaires (Quality of life, absenteeism, health care utilization).	The technology and the linked program allowed selection and targeting of teenagers who were most likely to benefit from a face-to-face encounter with their specialist. The value proposition of the technology was clear, with a distinct benefit for patients and an affordable service model, but health providers had plausible personal reasons to resist (double data entry). The organization was not yet ready for the innovation, as it required a shift to new ways of working. Dutch health insurers agreed that screen-to-screen consultations will be reimbursed at a rate equivalent to face-to-face consultations. The technology was considered easy to adapt and evolve over time to meet the needs of its users.

^a^Results [27,34-38,40-43] or conclusion [39] as described in the abstracts of the included studies.

^b^HIE: Health information exchange.

^c^HDIM: Home Dietary Intake Monitoring.

^d^ICBT: internet cognitive behavior therapy.

^e^COPD: chronic obstructive pulmonary disease.

^f^CVD: cardiovascular disease.

^g^IBD: inflammatory bowel disease.

### Indicators Organized by (Sub)themes of the SPO Framework

#### Overview

**Figure 5:**
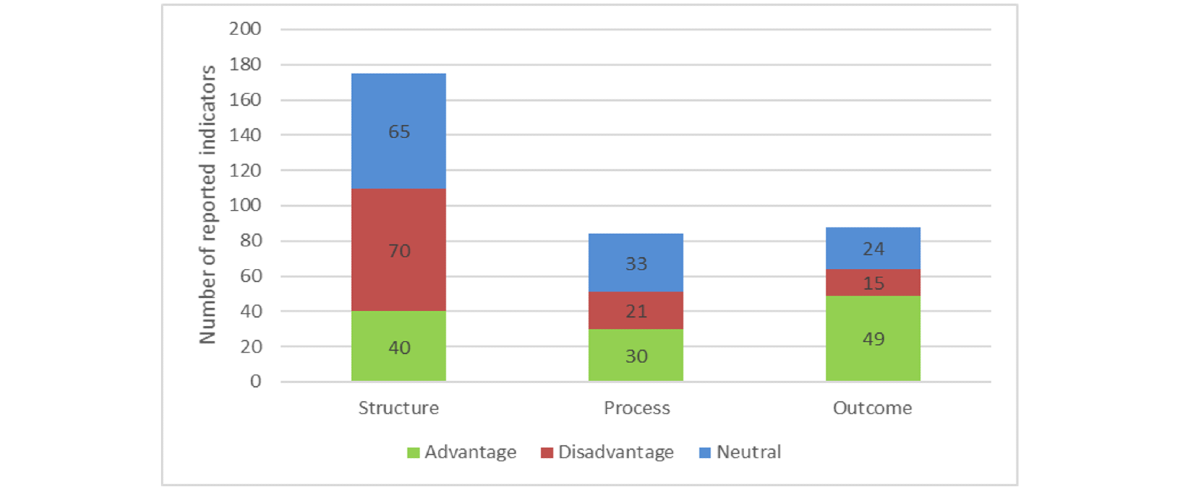
Number of indicators reported in the structure, process, and outcome categories. Advantage: in the structure and process categories, advantage indicates a positive effect on the integration. In the outcome category, it indicates a positive effect on the outcome. Disadvantage: in the structure and process categories, disadvantage indicates a negative effect on the integration. In the outcome category, it indicates a negative effect on the outcome. Neutral: Indicator was neither an advantage nor a disadvantage.

In total, an indicator was reported 347 times: 175 times in the structure category, 84 times in the process category, and 88 times in the outcome category. Of the 347 indicators, 111 were unique indicators (Multimedia Appendix 3)*.* In the structure category, most indicators were labeled as neutral (65/175, 37.1%) or as a disadvantage (70/175, 40%). In the process category, most indicators were labeled as an advantage (30/84, 36%) or neutral (33/84, 39%). In the outcome category, the indicators were mostly classified as a realized advantage (49/88, 56%), as shown in Figure 5.

Table 4 shows the total distribution of the indicators organized by themes and subthemes of the structure, process, and outcome categories and the extent to which it was reported as an advantage, disadvantage, or neutral to the integration of eHealth and its outcome in regular health care. The themes and subthemes containing the most reported indicators are described below.

**Table 4:** Distribution of the indicators according to the themes and subthemes of the structure, process, and outcome categories.

Category, theme, and subtheme			Advantage (n)	Disadvantage (n)	Neutral (n)	Source
**Structure (n=175)**						
	**Inner setting (n=51)**					
		Support of primary process (n=34)	7	13	14	[27,34,37-43]
		Culture and leadership (n=17)	7	9	1	[27,34,37,40]
	**Health care professional (n=28)**					
		Skills (n=8)	4	0	4	[27,36,38,40,41,43]
		Attitude (n=20)	8	8	4	[27,34-41,43]
	**Care receiver (n=40)**					
		Daily life (n=18)	3	8	7	[27,34-39]
		Baseline characteristics (n=22)	1	5	16	[34-39]
	**Technology (n=38**)					
		Usability and functionality (n=33)	8	17	8	[27,34-43]
		Interaction with EHR^a^ (n=5)	0	5	0	[35,37,38,41,43]
	**Outer setting (n=18**)					
		Finance and legislation (n=10)	0	2	8	[27,34,36,38-41]
		Involvement of stakeholders (n=8)	2	3	3	[27,38,43]
	Total structure		40	70	65	
**Process (n=84)**						
	**Health care actions (**n=**38)**					
		Workflow (n=18)	5	11	2	[27,34-39,41-43]
		Patient-centered (n=20)	7	0	13	[27,34-39,41,42]
	**Interpersonal actions (**n=**24)**					
		Personal (n=19)	11	3	5	[27,34-42]
		Shifting roles (n=5)	2	1	2	[34,36,42]
	**Process management (n=22)**					
		Quality improvement (n=11)	4	3	4	[27,34,38,40]
		Mistake-proofing (n=11)	1	3	7	[27,37-39,41-43]
	Total process		30	21	33	
**Outcome (n=88)**						
	**Health status (n=10)**					
		Clinical/functional (n=3)	1	0	2	[36,41,43]
		Intrapersonal (n=7)	6	0	1	[34,36,37,41,42]
	**Experience of care receiver** (n=**23)**					
		Satisfaction (n=16)	11	3	2	[34-38,42]
		Convenience (n=7)	7	0	0	[36,38,42]
	**Experience of health care professional** (n=**25**)					
		“What’s in it for me” (n=15)	9	2	4	[27,34,36-38,40,42]
		“What’s in it for them” (n=10)	10	0	0	[27,34,36-38,41-43]
	**Efficiency** **(n=30)**					
		Operations (n=27)	4	9	14	[34-43]
		Revenues (n=3)	1	1	1	[27,41,43]
	Total outcome		49	15	24	

^a^EHR: electronic health record.

#### Distribution of Indicators Within the Themes and Subthemes of the Structure Category

In the structure category, most indicators were reported in the inner setting (51/175, 29.1%), care receiver (40/175, 22.9%), and technology (38/175, 21.7%) themes. The indicators in the inner setting (n=22) and technology (n=23) themes were mainly classified as a disadvantage to the integration, whereas those in the care receiver theme (n=23) were mainly classified as neutral. Regarding the subthemes, most indicators were reported in the support of the primary process subtheme within the inner setting theme (34/175, 19.4%), the baseline characteristics subtheme within the care receiver theme (22/175, 12.6%), and the usability and functionality subtheme within the technology theme (33/175, 18.9%), as shown in Table 4.

#### Distribution of Indicators Within the Themes and Subthemes of the Process Category

Almost half of the indicators were organized within the health care actions theme (38/84, 45%), which were diversely reported as an advantage (n=13), disadvantage (n=11), and neutral (n=15). The subthemes with the most reported indicators were workflow (18/84, 21%), patient-centered (20/84, 24%), both within the health care actions theme, and the personal subtheme (19/84, 23%) within the interpersonal actions theme (Table 4).

#### Distribution of Indicators Within the Themes and Subthemes of the Outcome Category

In the outcome category, the most frequently reported indicators were from the efficiency theme (30/88, 34%), with advantages (n=5) reported for very few indicators. The “experiences” themes of care receivers and health care professionals together accounted for 55% (48/88), both predominated by advantages (n=37). The highest number of indicators were reported in the operations subtheme (n=27/88, 31%; Table 4).

#### Most Reported Indicators

An in-depth examination of the distribution of the indicators showed that the following four indicators were the most reported (ie, reported 10 times or more) among the included studies: “deployment of human resources” (n=11) of the inner setting theme in the structure category; “ease of use” (n=16) and “technical issue” (n=10), both belonging to the technology theme in the structure category; and “health logistics” (n=26) of the efficiency theme in the outcome category. An overview of all indicators is presented in Multimedia Appendix 3.

### Interactions Among Indicators Organized into Themes and Categories

#### Overview

**Figure 6:**
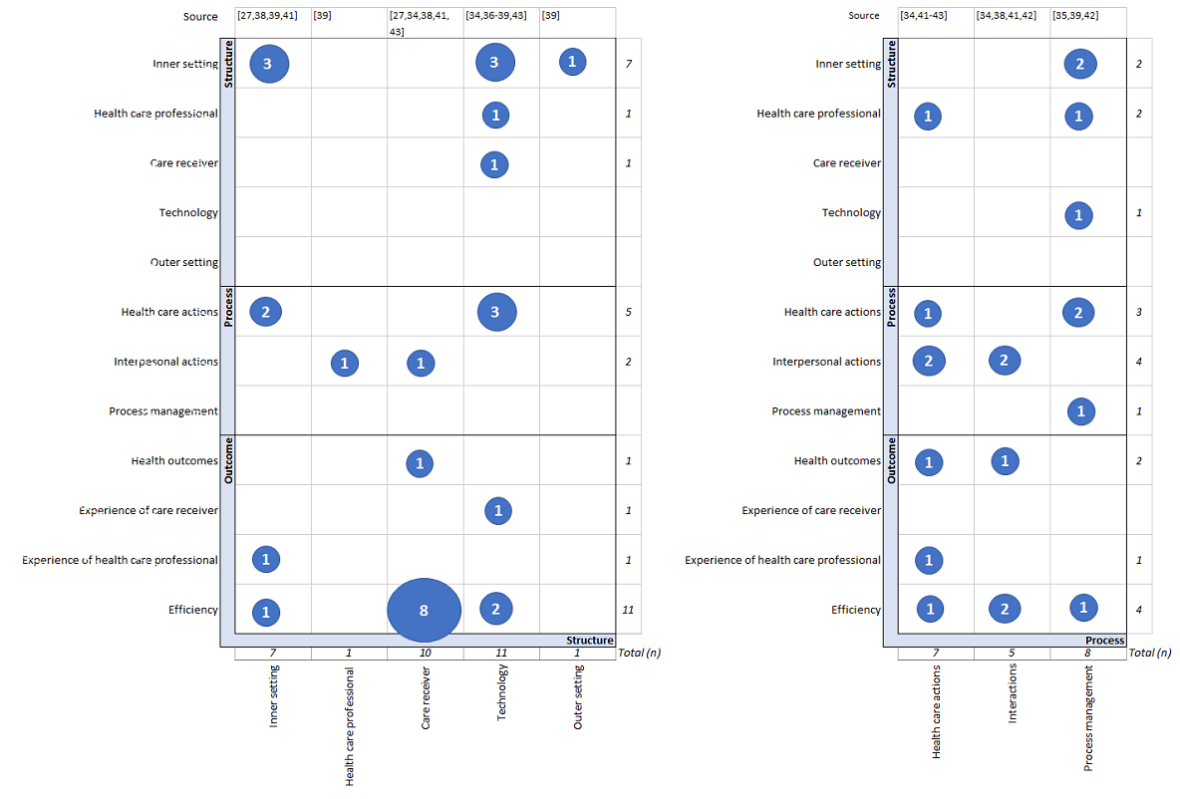
Interactions among indicators within themes and categories. The numbers within the blue circles represent the number of noted interactions among indicators within the themes. The x-axis represents the antecedent, and the y-axis represents the (intermediate) result.

Of the 11 included studies, 10 (91%) reported interactions among indicators organized by themes within the structure, process, and outcome categories. The most frequently reported interaction among indicators at the category level was between the structure and outcome categories (14 times). The most frequently reported interaction among indicators at the theme level was between the care receiver theme within the structure category and the efficiency theme within the outcome category (8 times), as shown in Figure 6.

#### Interactions With Themes in the Structure Category

All themes in the structure category contained indicators as an antecedent to, or as an intermediate result of other indicators. The inner setting (n=16), technology (n=12), and care receiver (n=11) themes represented the highest number of interactions with other themes. Inner setting was noted 7 times as an antecedent and 9 times as an intermediate result. Technology was noted 11 times as an antecedent and once as an intermediate result. Care receiver was noted 10 times as an antecedent and once as an intermediate result. The health care professional (n=3) and outer setting (n=1) themes were noted less frequently (Figure 6).

#### Interactions With Themes in the Process Category

In all themes in the process category, the indicators displayed interactions with indicators of other themes; specifically, health care theme (n=15), noted 7 times as an antecedent and 8 times as an intermediate result; interpersonal actions theme (n=11), 5 times as an antecedent and 6 times as an intermediate result; process management theme (n=9), 8 times as antecedent and once as an intermediate result (Figure 6).

#### Interactions With Themes in the Outcome Category

In the outcome category, the efficiency theme (n=15) contained most of the interacting indicators, all as an intermediate result. The other themes, including health status (n=3), experience of health care receiver (n=1), and experience of health care provider (n=2), were noted less frequently as (intermediate) results (Figure 6).

Examples of interactions among the indicators and the associated themes are illustrated in Textbox 2.

**Illustrations of reported interactions among indicators and their themes. Indicator names are written in italics as reported in the published studies (followed by the corresponding themes and categories in parentheses).**

- *Technical and usability issues* (technology theme, structure category) experienced by the health care professional negatively impacted the *engagement* and the *internal collaboration* (inner setting theme, structure category) [40] and the health care workflow by causing *extra steps* and *workarounds* (health care actions theme, process category) [37,41,43].
- *Technical and usability issues* (technology theme, structure category) experienced by the care receiver challenged the care receiver to *fit the application of eHealth into their daily lives* (care receiver theme, structure category) and caused increased *dropouts* (efficiency theme, outcome category) [34,39]. Conversely, one study [36] showed that technology that is *easy to use* (technology theme, structure category), can contribute positively to its application, and *fit into the patient’s daily life* (care receiver theme, structure category).
- Insufficient attention to the *patient’s burden* (care receiver theme, structure category), *health literacy* (care receiver theme, structure category), and whether the plan *fits into their daily life* (care receiver theme, structure category) caused *dropouts* (efficiency theme, outcome category) [36,37,39], and *nonadherence to care plans* (efficiency theme, outcome category) [34].
- *High workload* (inner setting theme, structure category) hindered the *incorporation of the application into daily practice* (inner setting theme, structure category) [40].
- *Lack of time* (inner setting theme, structure category) discouraged health care professionals from their *intention to (re)*use (experience health care professional theme, outcome category) [37] and health care professionals did not *experience an added value for themselves* (experience health care professional theme, outcome category) [37].
- *Communicated added value* (inner setting theme, structure category) on a corporate level positively influenced the *collective engagement* (inner setting theme, structure category) [40].
- *Guidelines on the work process* (process management, process category) made the *work process easier and faster for health care professionals* (health care actions theme, process category) but *limited the adaptability of the technology for certain recipients* (technology theme, structure category) [42].
- Limited *feedback about the quality of care* (process management theme, process category) made specialists feel *uncertain about the suitability of the technology* (health care professional theme, structure category) [38], whereas *sharing information* (process management theme, process category) to improve program efficiency allowed the program *to be a part of the workflow* (health care actions theme, process category) [38].
- *Face-to-face contact* (health care actions theme, process category) benefitted the *personal connection between care receiver and professional* (interpersonal actions theme, process category) and the *engagement of the care receiver with the treatment* (interpersonal actions theme, process category) [34].
- *Personal assistance* (health care actions theme, process category) and *personalized therapy* (health care actions theme, process category) increased the *usage of the intervention by the care receiver* (efficiency theme, outcome category) [39].
- *Personalized therapy* (health care actions theme) also increased the *satisfaction of the care receiver* (experience of care receiver theme, outcome category) [36].
- *Exceptions to the operational process* (health care actions theme, process category) were made too often, such as *providing extra support to patients* (health care actions theme, process category), or *providing less care* (health care actions theme, process category), *creating new administrative workarounds* (health care actions theme, process category) caused by *technical issues* (technology theme, structure category) [35,37,38,41,43] or *high workloads* (inner setting theme, structure category) [27].
- An increase in *questioning* by professionals (interpersonal actions theme, process category) made carers feel more *engaged and knowledgeable* (health status theme, outcome category) [42].
- Recipients’ *detailed input* (interpersonal actions theme, process category) on the assignments enabled professionals *to empathize with their situation* and *focus on their feedback* (interpersonal actions theme, process category) [34].

## Discussion

### Principal Findings

This literature review analyzed how eHealth can be organized optimally by using the Donabedian SPO framework. General organizational developments were identified, regardless of the type of illness, setting, or the eHealth application used. A review of the literature of selected cases highlighted three important findings. First, the role of the care recipient needs to be incorporated into the organizational structure and daily care process. Second, the technology must be well attuned to the structure of the organization and daily care process. Third, the deployment of the human resources to the daily processes needs to be aligned with the desired end results. Not adhering to these points could negatively affect the organization, daily process, or the end results. Findings from this research using the Donabedian framework corresponds to the conclusions of other studies using different research methodologies, which is explained below.

First, the SPO analysis showed that the care recipient plays a crucial role in the successful integration of eHealth. Patient-centered interaction and communication are important, to activate patients in managing their health care and to improve health outcomes in the application of eHealth [5,31,44-46]. Kuipers et al [44] and Rathert [45] demonstrated with systematic literature reviews that patient-centered care and co-creation are positively associated with the physical and social well-being of patients and with satisfaction of patients and health care professionals. These findings are in line with the review of Wildevuur and colleagues [46], demonstrating that organizations that are more patient centered with eHealth interventions achieve better outcomes with regard to patient health and quality of life. Although most health care professionals embrace more patient involvement and engagement, delegating power and responsibilities could be a challenge for health care professionals’ authority [47,48]. Another important issue is knowing who the customers are, what they want, and how the customer’s demand is answered [49]. A previous study reported that eHealth is not suitable for all care receivers [18,50]. Therefore, identifying who benefits most from which kind of therapy is an essential addition to the screening process, and it could lead to more effective targeting and resourcing [51]. Furthermore, insufficiently incorporating the patients’ family, work, and life goals into care plans will likely result in dropouts or nonadherence to care plans [50].

The second noteworthy finding is the essential role of excellent technology in the integration of eHealth. The way technology is set up has an influence on the working environment of health care professionals [52]. Inflexibility and complexity of the technology comes at the expense of effective daily processes and their quality [53,54]. Several studies demonstrated that the adaptability of eHealth technologies to fit to the local context, its ease of use, and its integration into clinical workflow benefit the users’ acceptance and meaningful use [22,55,56]. This was also reflected in the early phase of the COVID-19 pandemic, where rapid scalable technologies were the easiest to use and quickly implementable [53]. However, the health care system continued to face challenges in adopting digital technology after the first emergency phase of the COVID-19 pandemic, due to inadequate information and communication technology infrastructure and a bad fit of the technology into the clinical workflow that is primarily designed for face-to-face care [53]. Granja et al [54] demonstrated that the application of eHealth is often not fitted to the existing workflow due to time and space constraints and breaking of traditions. Although eHealth is seen as an innovative solution for alleviating the increasing burden for health care professionals [2], it could have a counterproductive effect on the working conditions for employees if the technology is not properly adapted to the structure and processes [57,58]. Third, integrating eHealth into a health care organization requires adjustments of the care processes and utilization of the human resources, with appropriate process monitoring. Working with eHealth also poses logistical challenges; for example, a clear understanding is needed of the expected achievements, processes, and staffing requirements in order to bring about changes and create new capabilities [59]. Vissers and De Vries [49] pointed out that it is necessary to know how the logistical capacities should be assigned to the process, how the processes are measured, and who is responsible for the management of the process. Changes in the workflow are inevitable and necessary for eHealth interventions to be successful [54]. However, integrating eHealth technology into daily care processes is complex, and it needs coordination and process communication [19]. For example, a living laboratory experiment conducted over 3 years with patients, health care professionals, enterprises, and researchers to accelerate the integration of eHealth in daily practice showed that workflow, responsibilities, and roles needed to change, but health care professionals did not know how to approach this and had difficulties in integrating eHealth into their daily care processes [18].

### Strength and Limitations

The strengths of this research are that international studies were included and represented a wide range of patient groups and settings. The findings were representative for the included studies, and they were not dependent of the study design, disease, target population, setting, or type or function of the eHealth application used. The wide range of settings of the included studies is supportive of a broader application of the present study’s findings. In the *Methods*, we stated that there is no clear consensus on what constitutes as *good eHealth* and how it is best organized [3,19]. Nevertheless, we believe that our findings make a significant contribution to improving the integration of eHealth in regular health care by identifying the most common indicators in the organization’s structure, processes, and outcomes. Thus, this research contributes to a new model for integrating organizational, health, and social factors.

A limitation of this study is that the health outcomes were rarely mentioned in this review. We hypothesized that this is because the main method used in the included studies was process evaluation. Therefore, although the health outcomes played a major role in earlier RCTs, this was not the case in process evaluation studies. The included studies did not define clear standards for the indicators to determine their quality. However, an indicator only becomes meaningful if a standard is specified [60,61]. There are also limitations in the selection procedure. The interrater reliability was not calculated. Due to this complex, broad topic, the predefined inclusion and exclusion criteria were sharpened at the time of selection. It was an iterative process, with a lot of consultation and coordination. In the process, full consensus was reached for all inclusion and exclusion criteria for selection at each step of the research. Another limitation is the classification of indicators into subthemes and themes at the discretion of the authors. It is conceivable that different classifications would reach different conclusions. Yet, the conclusions of each included study fit with the overall conclusion; therefore, the chance of this bias seems to be small. However, the findings of this literature review are dependent on the results of the included studies and may be subject to publication bias. Even though the included publications contain either positive or negative results (eg, a failed randomized trial [35] or interventions with no or less impact [40,43]), a chance of publication bias cannot be precluded automatically [62,63].

It is also noted that the Donabedian framework itself was designed before the introduction of eHealth and may not include the latest prevailing ideas on the organization of health care. For this reason, the model has been adapted in order to represent eHealth. By doing so, an attempt has been made to reduce the limitation as far as possible. Nevertheless, this literature review confirmed that it is still useful to analyze what contributes to the successful integration of eHealth into traditional health care. Additionally, there are other reputable models for evaluating eHealth interventions, such as the nonadoption, abandonment, scale-up, spread, sustainability (NASSS) framework [20]; Consolidated Framework for Implementation Research (CFIR) [64]; and the holistic framework to improve the uptake and impact of eHealth technologies [19]. These models describe the different phases from the design of the intervention to its adoption and implementation. This literature review focused on quality improvement of the way eHealth is organized, that has already passed the initial phase (of design and adoption). The Donabedian framework covers all relevant aspects for sustaining the integration of eHealth into health care and the interrelations of organization’s structure, processes, and outcomes, as well as integrating these aspects with human and social factors, after the adoption and uptake phase of eHealth.

### Conclusions

For optimal integration of eHealth into health care, the following main principles should be considered and approached simultaneously. First, the role of the care recipient needs to be incorporated in the organizational structure and daily care process. Second, the technology must be well attuned to the structure of the organization and daily care process. Third, the deployment of human resources to the care process needs to be aligned with the desired end results.

Thus far, no study has presented a complete overview of the successful and effective organization of eHealth. Therefore, it is desirable to supplement this research with knowledge from other sources, such as in-depth research into the experiences from different perspectives, as this can help us to obtain a complete overview of how eHealth can be successfully integrated into health care organizations.

## Appendix

Multimedia Appendix 1 Search strategy.

Multimedia Appendix 2 Explanatory notes on structure, process, and outcomes, and the (sub)themes.

Multimedia Appendix 3 Unique reported indicators.

## Abbreviations

CFIR: Consolidated Framework for Implementation Research
MMAT: Mixed Method Appraisal Tool
NASSS: nonadoption, abandonment, scale-up, spread, sustainability
NICE: National Institute for Health and Care Excellence
RCT: randomized controlled trial
PRISMA: Preferred Reporting Items for Systematic Reviews and Meta-analyses
SPO: Structure-Process-Outcome
