# Supplementary material for: The Challenge of Integrating eHealth Into Health Care: Systematic Literature Review of the Donabedian Model of Structure, Process, and Outcome
Source: J Med Internet Res. 2021 May 10;23(5):e27180. doi: 10.2196/27180 (PMC8145079; doi:10.2196/27180)
Supplement: Multimedia Appendix 1 [file jmir_v23i5e27180_app1.doc]

**APPENDIX 1: SEARCH STRATEGY**

How are structure indicators, process indicators related to ehealth and blended care outcomes indicators?

In the title and abstract we search for structure* indicators* or process* indicators* or outcomes* indicators and [blended care or ehealth* or telehealth*]:

structure* indicators* or process* indicators* or outcomes* indicators* and [blended care or ehealth* or telehealth*]:

("structure indicators" OR "process indicators" OR "outcomes indicators" OR "value proposition" OR "structure indicator" OR "process indicator" OR "outcomes indicator" OR "value propositions") AND ("blended care" OR ehealth* OR telehealth*)

Databases PubMed, EMBASE, Web of Science, Cochrane, Emcare:

**(**(((("structure"[ti] OR "structures"[ti]) AND ("process"[tiab] OR "processes"[tiab])) OR (("structure"[tiab] OR "structures"[tiab]) AND ("process"[ti] OR "processes"[ti]))) AND ("Outcome Assessment (Health Care)"[Mesh] OR "outcome"[tw] OR "outcomes"[tw]) AND (app[tw] OR apps[tw] OR Cell Phone[tw] OR Cell Phones[tw] OR cellular phone[tw] OR cellular phones[tw] OR computer application*[tw] OR computer assisted therapy[tw] OR computer assisted intervention[tw] OR computer assisted interventions[tw] OR Computer Mediated Communication[tw] OR Computer Mediated Communications[tw] OR computer-assisted instruction[tw] OR computer-assisted therapy[tw] OR computer-assisted[tw] OR digital health[tw] OR econsult*[tw] OR e-consult*[tw] OR ehealth[tw] OR e-health[tw] OR electronic communication*[tw] OR Electronic Learning[tw] OR Electronic Mail[mesh] OR Electronic Mail[tw] OR email*[tw] OR e-mail*[tw] OR information technology[tw] OR Internet[mesh] OR internet[tw] OR ipad*[tw] OR ipad[tw] OR iphon*[tw] OR mhealth[tw] OR m-health[tw] OR mobile health[tw] OR mobile*[tw] OR mobile[tw] OR multimedia[tw] OR online therapy[tw] OR personal digital assistant[tw] OR phone[tw] OR phones[tw] OR Reminder Device[tw] OR Reminder Devices[tw] OR reminder message[tw] OR reminder messages[tw] OR Reminder System[tw] OR Reminder Systems[mesh] OR Reminder Systems[tw] OR remote care[tw] OR remote communication[tw] OR remote computer[tw] OR remote computers[tw] OR "Remote Consultation"[mesh] OR remote consultation[tw] OR remote health care[tw] OR remote healthcare[tw] OR remote monitoring[tw] OR remote system[tw] OR remote systems[tw] OR remote technologies[tw] OR remote technology[tw] OR remote[tw] OR short message service[tw] OR smart phone[tw] OR smart technol*[tw] OR smart technology[tw] OR Smartphone[tw] OR Smartphones[tw] OR SMS[tw] OR social network*[tw] OR social network[tw] OR tablet*[tw] OR tele health[tw] OR telecare[tw] OR tele-care[tw] OR telecommunication*[tw] OR Telecommunications[mesh:noexp] OR teleconsult*[tw] OR teleconsultation[tw] OR telehealth[tw] OR tele-health[tw] OR telemed*[tw] OR Telemedicine[mesh:noexp] OR telemedicine[tw] OR telemonitoring[tw] OR tele-monitoring[tw] OR telenurs*[tw] OR telenursing[tw] OR telephon*[tw] OR Telephone[mesh] OR Telerehabilitation[mesh] OR telerehabilitation[tw] OR text messag*[tw] OR Text Messaging[tw] OR texting[tw] OR Therapy, computer-assisted[mesh:noexp] OR virtual community[tw] OR Virtual Reality[mesh] OR Virtual Reality[tw] OR wearable technologies[tw] OR wearable technology[tw] OR web access[tw] OR web application[tw] OR web applications[tw] OR web portal*[tw] OR web[ti] OR webapp*[tw] OR webbased[tw] OR web-based[tw] OR webcast*[tw] OR Webcasts as Topic[mesh] OR webpage[tw] OR webpages[tw] OR website[tw] OR websites[tw] OR blended care[tw] OR blended intervention[tw] OR blended interventions[tw] OR blended e health[tw] OR blended[tw])) **OR** (((("structure"[tw] OR "structures"[tw]) AND ("process"[tw] OR "processes"[tw]))) AND ("Outcome Assessment (Health Care)"[Mesh] OR "outcome"[tw] OR "outcomes"[tw]) AND (app[ti] OR apps[ti] OR Cell Phone[ti] OR Cell Phones[ti] OR cellular phone[ti] OR cellular phones[ti] OR computer application*[ti] OR computer assisted therapy[ti] OR computer assisted intervention[ti] OR computer assisted interventions[ti] OR Computer Mediated Communication[ti] OR Computer Mediated Communications[ti] OR computer-assisted instruction[ti] OR computer-assisted therapy[ti] OR computer-assisted[ti] OR digital*[ti] OR digital[ti] OR digital health[ti] OR econsult*[ti] OR e-consult*[ti] OR ehealth[ti] OR e-health[ti] OR electronic communication*[ti] OR Electronic Learning[ti] OR Electronic Mail[majr] OR Electronic Mail[ti] OR email*[ti] OR e-mail*[ti] OR information technology[ti] OR Internet[majr] OR internet[ti] OR ipad*[ti] OR ipad[ti] OR iphon*[ti] OR mhealth[ti] OR m-health[ti] OR mobile health[ti] OR mobile*[ti] OR mobile[ti] OR multimedia[ti] OR on line[ti] OR online therapy[ti] OR online[ti] OR on-line[ti] OR personal digital assistant[ti] OR phone[ti] OR phones[ti] OR Reminder Device[ti] OR Reminder Devices[ti] OR reminder message[ti] OR reminder messages[ti] OR Reminder System[ti] OR Reminder Systems[majr] OR Reminder Systems[ti] OR remote care[ti] OR remote communication[ti] OR remote computer[ti] OR remote computers[ti] OR "Remote Consultation"[majr] OR remote consultation[ti] OR remote health care[ti] OR remote healthcare[ti] OR remote monitoring[ti] OR remote system[ti] OR remote systems[ti] OR remote technologies[ti] OR remote technology[ti] OR remote[ti] OR short message service[ti] OR smart phone[ti] OR smart technol*[ti] OR smart technology[ti] OR Smartphone[ti] OR Smartphones[ti] OR SMS[ti] OR social network*[ti] OR social network[ti] OR tablet*[ti] OR tele health[ti] OR telecare[ti] OR tele-care[ti] OR telecommunication*[ti] OR Telecommunications[majr:noexp] OR teleconsult*[ti] OR teleconsultation[ti] OR telehealth[ti] OR tele-health[ti] OR telemed*[ti] OR Telemedicine[majr:noexp] OR telemedicine[ti] OR telemonitoring[ti] OR tele-monitoring[ti] OR telenurs*[ti] OR telenursing[ti] OR telephon*[ti] OR Telephone[majr] OR Telerehabilitation[majr] OR telerehabilitation[ti] OR text messag*[ti] OR Text Messaging[ti] OR texting[ti] OR Therapy, computer-assisted[majr:noexp] OR virtual community[ti] OR Virtual Reality[majr] OR Virtual Reality[ti] OR wearable technologies[ti] OR wearable technology[ti] OR web access[ti] OR web application[ti] OR web applications[ti] OR web portal*[ti] OR web[ti] OR webapp*[ti] OR webbased[ti] OR web-based[ti] OR webcast*[ti] OR Webcasts as Topic[majr] OR webpage[ti] OR webpages[ti] OR website[ti] OR websites[ti] OR blended care[ti] OR blended intervention[ti] OR blended interventions[ti] OR blended e health[ti] OR blended[ti])) **OR** (("structure indicators"[tw] OR "process indicators"[tw] OR "structure indicator"[tw] OR "process indicator"[tw]) AND ("Outcome Assessment (Health Care)"[Mesh] OR "outcome"[tw] OR "outcomes"[tw]) AND (app[tw] OR apps[tw] OR Cell Phone[tw] OR Cell Phones[tw] OR cellular phone[tw] OR cellular phones[tw] OR computer application*[tw] OR computer assisted therapy[tw] OR computer assisted intervention[tw] OR computer assisted interventions[tw] OR Computer Mediated Communication[tw] OR Computer Mediated Communications[tw] OR computer-assisted instruction[tw] OR computer-assisted therapy[tw] OR computer-assisted[tw] OR digital*[tw] OR digital[tw] OR digital health[tw] OR econsult*[tw] OR e-consult*[tw] OR ehealth[tw] OR e-health[tw] OR electronic communication*[tw] OR Electronic Learning[tw] OR Electronic Mail[mesh] OR Electronic Mail[tw] OR email*[tw] OR e-mail*[tw] OR information technology[tw] OR Internet[mesh] OR internet[tw] OR ipad*[tw] OR ipad[tw] OR iphon*[tw] OR mhealth[tw] OR m-health[tw] OR mobile health[tw] OR mobile*[tw] OR mobile[tw] OR multimedia[tw] OR on line[tw] OR online therapy[tw] OR online[tw] OR on-line[tw] OR personal digital assistant[tw] OR phone[tw] OR phones[tw] OR Reminder Device[tw] OR Reminder Devices[tw] OR reminder message[tw] OR reminder messages[tw] OR Reminder System[tw] OR Reminder Systems[mesh] OR Reminder Systems[tw] OR remote care[tw] OR remote communication[tw] OR remote computer[tw] OR remote computers[tw] OR "Remote Consultation"[mesh] OR remote consultation[tw] OR remote health care[tw] OR remote healthcare[tw] OR remote monitoring[tw] OR remote system[tw] OR remote systems[tw] OR remote technologies[tw] OR remote technology[tw] OR remote[tw] OR short message service[tw] OR smart phone[tw] OR smart technol*[tw] OR smart technology[tw] OR Smartphone[tw] OR Smartphones[tw] OR SMS[tw] OR social network*[tw] OR social network[tw] OR tablet*[tw] OR tele health[tw] OR telecare[tw] OR tele-care[tw] OR telecommunication*[tw] OR Telecommunications[mesh:noexp] OR teleconsult*[tw] OR teleconsultation[tw] OR telehealth[tw] OR tele-health[tw] OR telemed*[tw] OR Telemedicine[mesh:noexp] OR telemedicine[tw] OR telemonitoring[tw] OR tele-monitoring[tw] OR telenurs*[tw] OR telenursing[tw] OR telephon*[tw] OR Telephone[mesh] OR Telerehabilitation[mesh] OR telerehabilitation[tw] OR text messag*[tw] OR Text Messaging[tw] OR texting[tw] OR Therapy, computer-assisted[mesh:noexp] OR virtual community[tw] OR Virtual Reality[mesh] OR Virtual Reality[tw] OR wearable technologies[tw] OR wearable technology[tw] OR web access[tw] OR web application[tw] OR web applications[tw] OR web portal*[tw] OR web[ti] OR webapp*[tw] OR webbased[tw] OR web-based[tw] OR webcast*[tw] OR Webcasts as Topic[mesh] OR webpage[tw] OR webpages[tw] OR website[tw] OR websites[tw] OR blended care[tw] OR blended intervention[tw] OR blended interventions[tw] OR blended e health[tw] OR blended[tw])) **OR** (("disease management"[majr] OR "disease management"[ti] OR "self management"[ti] OR "Health Services Accessibility"[majr]) AND ("adoption"[ti] OR implement*[ti] OR "incorporating"[ti] OR "use"[ti] OR "usage"[ti]) AND (app[ti] OR apps[ti] OR Cell Phone[ti] OR Cell Phones[ti] OR cellular phone[ti] OR cellular phones[ti] OR computer application*[ti] OR computer assisted therapy[ti] OR computer assisted intervention[ti] OR computer assisted interventions[ti] OR Computer Mediated Communication[ti] OR Computer Mediated Communications[ti] OR computer-assisted instruction[ti] OR computer-assisted therapy[ti] OR computer-assisted[ti] OR digital*[ti] OR digital[ti] OR digital health[ti] OR econsult*[ti] OR e-consult*[ti] OR ehealth[ti] OR e-health[ti] OR electronic communication*[ti] OR Electronic Learning[ti] OR Electronic Mail[majr] OR Electronic Mail[ti] OR email*[ti] OR e-mail*[ti] OR information technology[ti] OR Internet[majr] OR internet[ti] OR ipad*[ti] OR ipad[ti] OR iphon*[ti] OR mhealth[ti] OR m-health[ti] OR mobile health[ti] OR mobile*[ti] OR mobile[ti] OR multimedia[ti] OR on line[ti] OR online therapy[ti] OR online[ti] OR on-line[ti] OR personal digital assistant[ti] OR phone[ti] OR phones[ti] OR Reminder Device[ti] OR Reminder Devices[ti] OR reminder message[ti] OR reminder messages[ti] OR Reminder System[ti] OR Reminder Systems[majr] OR Reminder Systems[ti] OR remote care[ti] OR remote communication[ti] OR remote computer[ti] OR remote computers[ti] OR "Remote Consultation"[majr] OR remote consultation[ti] OR remote health care[ti] OR remote healthcare[ti] OR remote monitoring[ti] OR remote system[ti] OR remote systems[ti] OR remote technologies[ti] OR remote technology[ti] OR remote[ti] OR short message service[ti] OR smart phone[ti] OR smart technol*[ti] OR smart technology[ti] OR Smartphone[ti] OR Smartphones[ti] OR SMS[ti] OR social network*[ti] OR social network[ti] OR tablet*[ti] OR tele health[ti] OR telecare[ti] OR tele-care[ti] OR telecommunication*[ti] OR Telecommunications[majr:noexp] OR teleconsult*[ti] OR teleconsultation[ti] OR telehealth[ti] OR tele-health[ti] OR telemed*[ti] OR Telemedicine[majr:noexp] OR telemedicine[ti] OR telemonitoring[ti] OR tele-monitoring[ti] OR telenurs*[ti] OR telenursing[ti] OR telephon*[ti] OR Telephone[majr] OR Telerehabilitation[majr] OR telerehabilitation[ti] OR text messag*[ti] OR Text Messaging[ti] OR texting[ti] OR Therapy, computer-assisted[majr:noexp] OR virtual community[ti] OR Virtual Reality[majr] OR Virtual Reality[ti] OR wearable technologies[ti] OR wearable technology[ti] OR web access[ti] OR web application[ti] OR web applications[ti] OR web portal*[ti] OR web[ti] OR webapp*[ti] OR webbased[ti] OR web-based[ti] OR webcast*[ti] OR Webcasts as Topic[majr] OR webpage[ti] OR webpages[ti] OR website[ti] OR websites[ti] OR blended care[ti] OR blended intervention[ti] OR blended interventions[ti] OR blended e health[ti] OR blended[ti]))**)** NOT ("Animals"[mesh] NOT "Humans"[mesh])
