# Supplementary material for: The Challenge of Integrating eHealth Into Health Care: Systematic Literature Review of the Donabedian Model of Structure, Process, and Outcome
Source: J Med Internet Res. 2021 May 10;23(5):e27180. doi: 10.2196/27180 (PMC8145079; doi:10.2196/27180)
Supplement: Multimedia Appendix 2 [file jmir_v23i5e27180_app2.docx]

**APPENDIX 2**

**Explanatory notes on structure, process, outcomes and the (sub)themes**

**Textbox 2**

| STRUCTURE  **Inner setting**: The administrative structure and operations in the institute   - *Support of primary process*: The created/facilitated conditions to provide care, e.g. training skills, available resources, workload balance, supply of information - *Culture & leadership*: The specific collection of values and norms that are shared by people within an organisation and the internal collaboration and collective engagement. Leadership relates to a leadership that inspires the organisation with the values, the way they communicate these values, but also the traditional leadership, e.g. setting priorities, strategic goals, etc.   **Healthcare professionals**: Characteristics of the healthcare providers   - *Skills*: Competence with treatment aspects, technology, computer - *Attitude*: Confidence and/or comfort with the intervention and, or in the patient’s competence to use; willingness to use/learn; belief in program’s value   **Care receiver:** Characteristics of the care receiver   - *Daily life*: Household and lifestyle; access to technology, insurance cover, fit with daily life, (lack) of time - *Baseline characteristics*: Age, gender, SES, skills, attitude (e.g. believes in program’s value), quality of life, cognitive/physical functioning, therapy compliance   **Technology:** The adequacy of the facility and technological equipment to provide eHealth   - *Usability and functionality:* Its ease of use, technical performance, quality of the audiovisual aspects - *Interaction with EHR*: It interacts with the Electronic Health Record in use.   **Outer Setting**: The administrative structure and operations in the environment outside the institute (government/policies/regulations/network)   - *Finance & Legislation*: Policy context, regulatory, reimbursement - *Involvement of stakeholders*: Collaboration of external stakeholders; fit with the community needs; external communication |
| --- |

**Textbox 3**

| PROCESS  **Healthcare actions:** The actual healthcare which is given and received   - *Workflow*: The steps and time the actual healthcare requires and the extent of integrating it into conventional healthcare; reduction of work, integration with workflow, (lack) of consultation time - *Patient-centred*: Creation of conditions in the workflow for patient-centred care. Personal assistance, personalised medicine/therapy, screening of patient’s eligibility   **Interpersonal actions:** Interactions between care receiver and health professionals   - *Personal*: Development of a therapeutic relationship and/or openness/compliance with the intervention. - *Shifting roles*: Shift in the power balance in the relationship; changing role of practice; refocus treatment elements   **Process management:** The action to improve the quality of the healthcare process in question   - *Quality improvement:* Monitoring and improvement activities (re-active) e.g. best practices, clinical feedback, continued development of guidelines - *Mistake-proofing*: error prevention activities (pro-active), e.g. notifications, (systematic) guidance in the work process, using guidelines |
| --- |

**Textbox 4**

| OUTCOME  **Health status:** The clinical, functional and intrapersonal health outcomes   - *Clinical/functional*: Clinical, functional outcomes e.g. vital values, pain reduction, performance of organs or joints. - *Intrapersonal:* Quality of live, self-efficacy, personal confidence   **Experience of care recipients:** Satisfaction and convenience   - *Satisfaction:* Attitude towards care received (trust, confidence, satisfaction) - *Convenience*: Reduced travel, increased access   **Experience of healthcare professionals:** Gains for job performance and gains for clients, according to the healthcare professional   - *"What's in it for me":* e.g. satisfied, intends to re-use, burdensome/demanding - *"What's in it for them":* Believes that is helpful for the care receiver, that the care receiver is satisfied, etc.   **Efficiency:** Business consequences of the healthcare is provided   - *Operations*: Operational performance; e.g. response time, number of contacts, performance according to protocol, drop-outs, reschedules, processing time, waiting time - *Revenues*: Costs, turnover |
| --- |
